# The effect of restored medial knee anatomy in total knee arthroplasty with the flexion first balancer technique on mid-flexion laxity and functional outcome

**DOI:** 10.1186/s12891-021-04869-3

**Published:** 2021-12-09

**Authors:** W. A. M. van Lieshout, I. van Oost, K. L. M. Koenraadt, L. H. G. J. Elmans, R. C. I. van Geenen

**Affiliations:** 1 Department of Orthopedic Surgery, Molengracht 21, Amphia Breda, 4818 CK The Netherlands; 2 Foundation for Orthopaedic Research, Care & Education (FORCE), Amphia Breda, The Netherlands

**Keywords:** Total knee replacement, Flexion first balancer, Stress radiographs, Mid-flexion laxity, Coronal laxity, Functional outcome

## Abstract

**Background:**

The Flexion First Balancer (FFB) technique for total knee arthroplasty (TKA) was developed to maintain the isometry of the medial collateral ligament (MCL) by restoring the medial anatomy of the knee. Inability to correct MCL isometry could hypothetically result in an increased mid-flexion laxity. The aim of the current study was to evaluate if the FFB technique results in improved functional outcome and less mid-flexion laxity compared to Measured Resection (MR).

**Methods:**

A cross-sectional study was performed comparing 27 FFB patients with 28 MR patients. Groups were matched for age, gender, BMI and ASA classification. All patient received the cruciate retained type, Vanguard Complete Knee System (Biomet Orthopedics, Warsaw, IN, USA). Stress X-rays of the knee with 30 degrees of flexion were made to assess varus-valgus laxity. Furthermore, three tests were conducted to asses functional outcome: a 6 min walk test, a stair climb test and quadriceps peak force measurements. Mean follow-up was respectively 2.6 (SD 0.4) and 3.9 years (SD 0.2).

**Results:**

The MR group showed a postoperative elevation in joint line in contrast to the FFB group, the mean difference between the two groups was 3 mm (*p* < 0.001). No differences in total laxity between the two groups was found. The FFB group showed a higher quadriceps peak force (1.67 (SD 0.55) N/BMI) in comparison with the MR group (1.38 (SD 0.48) N/BMI) (*p* < 0.05). All other outcome parameters were comparable between the two groups (p: n.s.). Correlation analysis showed a moderate negative correlation between joint line elevation and quadriceps peak force (r = − 0.29, *p* < 0.05).

**Conclusion:**

The FFB technique did not lead to less coronal laxity in the mid-flexion range compared to MR. Although peak quadriceps force was significantly higher for the FFB group no clinically relevant benefits could be identified for the patients with regards to functional outcome. Therefore, minor deviations in joint line seems to have no effect on functional outcome after TKA.

**Trial registration:**

ISRCTN, ISRCTN85351296. Registered 23 april 2021 - Retrospectively registered, https://www.isrctn.com/ISRCTN85351296

## Background

With the standard Measured Resection (MR) technique in total knee arthroplasty (TKA) the medial posterior condylar offset and joint line height are regularly not fully restored resulting in an elevation of the joint line [1]. For gap balancing techniques this is even slightly higher [2]. This elevation of the joint line hypothetically results in an increased mid-flexion laxity due to loss of isometry for the medial collateral ligament (MCL) [3, 4]. Restoring the medial joint line height to its pre-disease height is advocated to achieve a balanced knee [5].

The Flexion First Balancer (FFB) technique for TKA was developed to maintain the isometry of the MCL by restoring the medial anatomy of the knee [6]. Although in a previous report joint line reconstruction with FFB was achieved in contrast to MR, this did not result in better patient reported outcome measures (PROMs) [7], a correlation with functional outcome tests however can be expected [8].

In recent literature extra focus on flexion gap balancing during TKA is seen to achieve better performance [2, 9]. But while these previous studies focused on antero-posterior stability of the knee or patella tracking, the current study focused on medio-lateral stability which still is considered the key to achieve satisfactory results.

The aim of the current study was to investigate the effect of the FFB technique on postoperative coronal laxity in the mid-flexion range and functional outcome. Our hypothesis was that since the FFB technique restores the joint line to its pre-disease height this would result in less coronal mid-flexion laxity. Which, in term could result in better functional outcome compared to the standard MR technique due to increased stability in the knee [4, 5].

## Methods

### Study population

**Fig. 1:**
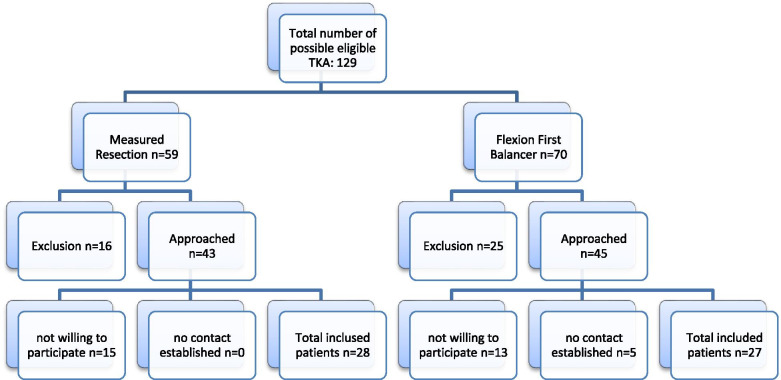
Flow chart study

In the present study patients who underwent knee replacement surgery via the FFB technique and were treated between September 2015 and November 2016 were included. The control group was composed out of a historical matched cohort of patients who were operated using the standard MR technique between September 2014 and July 2015. The MR group was matched to the FFB group based on age, gender, Body mass index (BMI) and American Society of Anesthesiologists (ASA) classification, using frequency matching. Patients were operated by two surgeons, using the FFB technique as well as the MR technique for TKA as standard care. In all patients a cruciate retained (CR) type, Vanguard Complete Knee System (Biomet Orthopedics, Warsaw, IN, USA) implant was used. Inclusion criteria for analysis were primary cruciate retaining TKA for Kellgren-Lawrence grade 3-4 osteoarthritis with at least 1 year of follow up. Exclusion criteria were ill health influencing functional tests (e.g. severe chronic obstructive pulmonary disease or congestive heart failure), degenerative diseases (e.g. rheumatoid arthritis) or development of severe osteoarthritis in other lower extremity joints that would influence functional test outcomes, complications requiring consecutive surgery, a hip replacement or contralateral TKA within the past year or previous knee surgery which could influence the laxity of the knee (MCL or LCL reconstruction/reefing). All patients gave written informed consent prior to the investigation. A total of 55 patients were included, 27 patients in the FFB group and 28 patients in the MR group resulting in 27 FFB knees and 28 MR knees included in this study (Fig. 1). Baseline characteristics regarding patients and type and severity of arthrosis were comparable between the matched groups (Table 1). The study protocol was evaluated and approved by the Dutch medical ethical committee MEC-U (protocol ID: NL65535.100.18).

**Table 1:** Baseline characteristics

	MR (***n*** = 28)	FFB (***n*** = 27)	***p***-value
**Age, mean (sd)**	67.0 (7.7)	66.4 (7.9)	N.S.
**Gender, no. male (%)**	11 (39.3)	15 (55.6)	N.S.
**BMI, mean (sd)**	30.2 (4.9)	29.2 (3.9)	N.S.
**ASA, no. (%)**			N.S.
**I**	5 (17.9)	6 (22.2)	
**II**	17 (60.7)	20 (74.1)	
**III-IV**	6 (21.4)	1 (3.7)	
**Follow up, mean (sd)**	3.9 yrs. (0.2)	2.6 yrs. (0.4)	N.A.
**Type of arthrosis (Medial/Lateral/Multi/PF)**	14 / 3 / 10 / 1	9 / 6 / 11 / 1	N.S
**FTA, mean (sd)**	3.1 (4.8)	3.0 (5.4)	N.S.
**Bone-loss**	2	6	N.S.

*Abbreviations*: *MR* measured resection, *FFB* Flexion First Balancer, *sd* standard deviation, *BMI* body mass index, *ASA* American Standardization Association, *PF* patella-femoral, *FTA* femoral tibial angle, *N.A*. not applicable, *N.S*. non-significant

For this cross-sectional study, patients were asked to visit our hospital for further evaluation. This consisted of extra radiographs to test mid-flexion laxity, three functional tests, and two questionnaires.

### Surgical technique FFB

This new technique for TKA was designed to enable the surgeon to reproducibly retain MCL isometry through preservation of the medial PCO and thereby reproduce the medial pre-disease joint line height. Because of the intact MCL isometry, mid-flexion instability presumably would not occur. The hypothesis for this new technique was that with retained MCL isometry and subsequent restoration of the medial joint line, less objective knee laxity would be present. This, in term, should result in better patient reported outcome, more subjective knee stability, and higher functional performance for these patients. For a detailed surgical technique description and preliminary results regarding outcome we refer to previously published works by W. van Lieshout et al. [6, 7].

### Radiological analysis

To determine mid-flexion laxity of the knee, three anterior-posterior radiographs of the knee were ordered. The knee was positioned in an angle of approximately 30° using a 15 cm high triangle cushion under the knee for all three radiographs. Firstly, a neutral radiograph was obtained. Subsequently, two radiographs were obtained while medial or lateral forces were applied to the knee. The direction of the X-rays were parallel to the tibia joint surface, centered on the middle of the femorotibial joint space. Varus and valgus stress were applied to the knee with a load of 15 Nm using the Telos device (Fa Telos, Medizinisch-Technische GmbH, Griesheim, Germany). Patients were in a supine position with leg muscles relaxed. For joint line measurements standard AP radiographs of the knee were used. Preoperatively, pre-disease (i.e. without cartilage loss) X-rays were analysed and compared with the 1-year postoperative X-rays. The femoral tibial angle (FTA) was determined to assess pre-operative alignment. For the preoperative FTA we used the last X-ray before surgery. Femoral-tibial angle was defined as the angle between the anatomical axis of the femur and the tibia in the anteroposterior view of the knee.

To determine changes in joint line level the adductor ratio was calculated with correction for cartilage depth as described in a previous study comparing FFB and MR for PROMs and outcome [7]. For the coronal mid-flexion laxity measurements the angle between a line through the distal femoral component condyles and a line through the tibia component was determined. This was done on the varus, valgus and neutral radiographs using the measurement tool within the radiographic JiveX software (Visus Technology Transfer GmbH, version 4.7.1.10, Bochem Germany). With this software, measurements with an accuracy of 0.1° were possible. Valgus laxity was defined as the difference in angles between the medial stress radiograph and the neutral radiograph, varus laxity as the difference between the lateral stress radiograph and the neutral radiograph. Total laxity was defined as the total of the varus and valgus laxity achievable in the knee. This method has previously successfully been used and described by Heesterbeek et al. [10] although real validation is lacking in literature it is frequently used. To assess the validity of the measurement itself we calculated the ICC by re-measuring the first 10 sets of radiographs (neutral, varus and valgus stress). This provided us with an ICC of respectively 0.991, 0.995 and 0.998.

### Outcome measures

During the visit, patients were asked to fill out the validated Dutch EQ-5D-5L and Lysholm questionnaire [11–14]. The first questionnaire evaluates (EQ-5D-5L) the quality of life experienced by the patient at the time of the survey. The second test (Lysholm) evaluates the knee stability experienced by the patient and the ability to perform daily living activities. The aim for these questionnaires was to evaluate if a more specific questionnaire on QOL and knee stability could detect differences between the two groups.

**Fig. 2:**
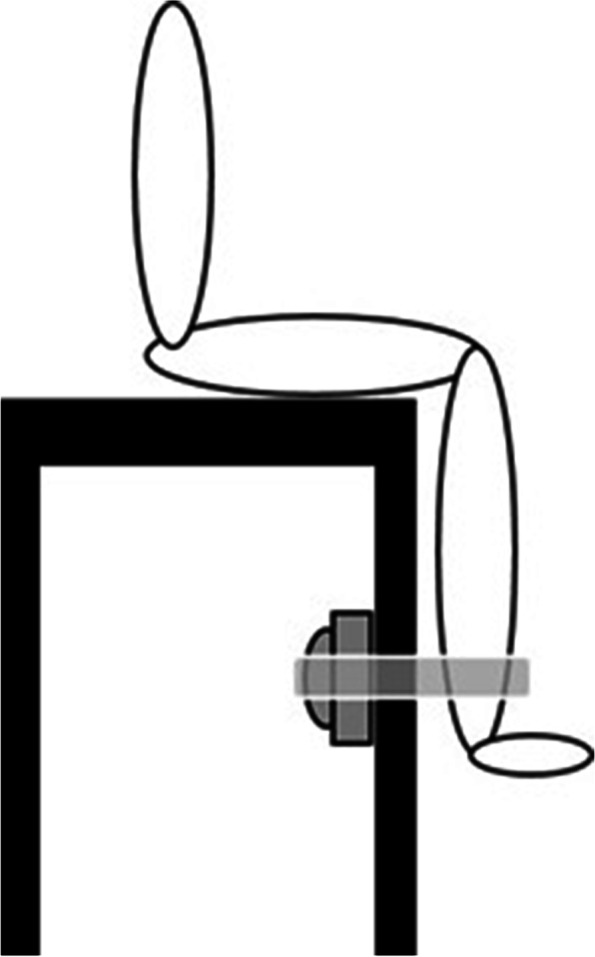
Experimental setup for the quadriceps force measurements. Patients were asked to sit on a table. The hand held dynamometer (represented by the dark gray element) was fixed at the posterior side of the table leg. The strap (represented by the light gray bar) was then positioned around the lower leg and the hand held dynamometer. The standard height of the strap was 5 cm above the medial malleolus

Finally, the patients were asked to perform three functional tests in the following fixed order: a timed stair climbing test (SCT), a 6-min walking test (6MWT) and a static quadriceps peak force test. These three test have been shown to correlate with passive mid-flexion laxity in a recent study [8]. The SCT was measured as the time necessary to ascend and descend a 10-step staircase as previously described [15]. The participants were instructed to complete the task as quickly and safely as possible. They were encouraged not to use the handrail unless necessary. The 6MWT was measured as the distance a participant could walk around a 25 m indoor track in 6 min. Patients were instructed to walk as far as possible in a safe manner. They were encouraged not to take any breaks or to use a walking aid unless this was necessary to complete the task [16, 17]. For the static quadriceps peak force test patients were asked to sit on the edge of a table with their knees in 90 degrees flexion holding the lower leg parallel to the table leg. The lower leg was then non-elastically strapped 5 cm above the medial malleolus to a hand-held dynamometer (HHD) meter which was positioned on the posterior side of the table leg (Fig. 2). Patients were instructed to hold their arms in front of their chest and apply maximum amount of knee extension for 5 s. This modified method of testing with a HHD has proven to be highly reliable [18]. The first test outcome was recorded as a trial followed by two recorded outcomes.

### Statistical analysis

The two quadriceps force measurements were averaged and divided by the BMI of the patient. Normally distributed continuous data were compared between the groups using unpaired samples T-tests, otherwise Mann-Whitney U Tests were used. Categorical data was compared between groups using Chi-square tests. To evaluate changes in outcome parameters over time (e.g. the adductor ratio) paired samples T-tests were used. Pearson’s correlation coefficients were determined to evaluate the association between joint line changes and laxity and between these parameters and the functional outcome parameters. Sample size calculations were based on mid-flexion stability as our primary outcome measure. Based on a clinical relevant difference of 0.5 mm, an SD of 0.5 mm, an alpha of 0.05 and a power of 0.95, two groups of 24 patients was needed [19]. To allow for possible exclusions we aimed to include a maximum of 30 patients per group. *P*-values below 0.05 were considered as significant. SPSS software package (version 25.0, IBM Corp., Armonk, NY, USA) was used to perform the statistical analyses.

## Results

Mean follow up for the 27 patients in the FFB group was 2.6 years (SD 0.4) and 3.9 (SD 0.2) years for the 28 patients in the MR group. All patients had at least 2 years follow up except for 1 FFB patients (15 months). The difference in technique between the MR and FFB technique with regard to joint line position was demonstrated by a significantly higher joint line after surgery in the MR group compared to the FFB group. I.e. the preoperative adductor ratio was comparable between the two groups (MR: 0.552 ± 0.033 vs. FFB: 0.554 ± 0.034, *p* = 0.80), but after surgery a significant difference between the groups was found in the delta score of the adductor ratio (MR:-0.014 ± 0.024 vs. FFB: 0.015 ± 0.021, *p* < 0.001), indicative of an absolute difference of 3 mm between the two groups. The adductor ratio in the MR group decreased significantly (*p* < 0.01) and in the FFB group a significant increase was found (*p* < 0.005).

**Fig. 3:**
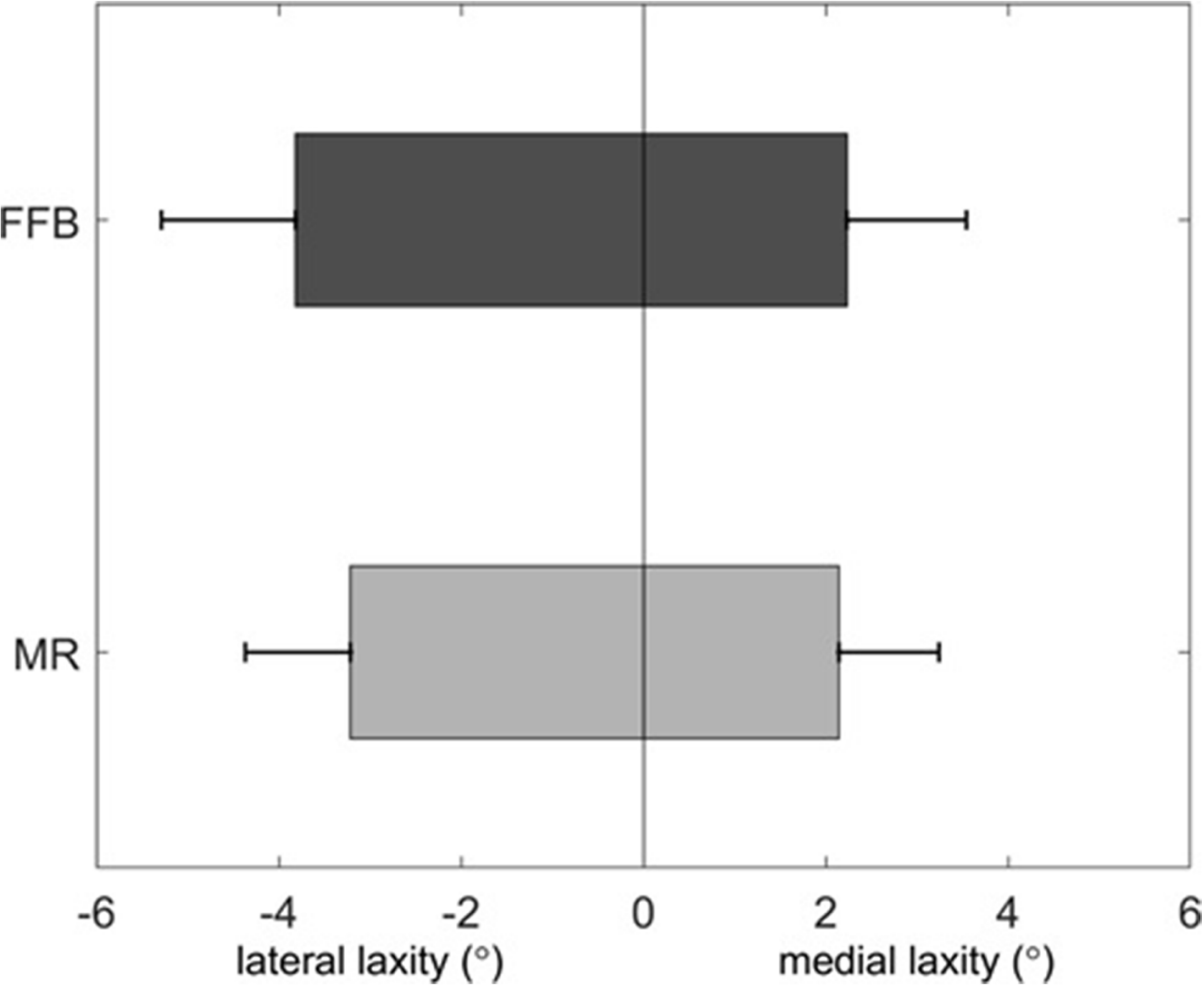
Medial and lateral mid-flexion laxity in the FFB vs MR group. The boxplots show the mean lateral and medial laxity in 30 degrees of knee flexion for the Flexion First Balancer (FFB) group and of the Measured Resection (MR) group. Together this shows the total laxity per group. Represented on the left side is the mean lateral laxity for FFB: 3.8° (SD 1.5°) and for MR: 3.2° (SD 1.2°). Represented on the right side is the mean medial laxity for FFB: 2.2° (SD 1.3°) and for MR: 2.1° (SD 2.1°). All data comparisons were non-significant

With respect to the primary outcome, no difference was seen in medial laxity (*p* = 0.78), lateral laxity (*p* = 0.10) and total laxity (*p* = 0.14) between the FFB group and the MR group (Fig. 3). The secondary outcomes demonstrated a significantly higher quadriceps peak force in the FFB group compared to the MR group (*p* = 0.03). In respect of the other functional tests (6MWT and SCT) and the questionnaires, no differences between the two groups were found (*p* = n.s.) (Table 2).

**Table 2:** Secondary outcome measures; functional test and questionnaires

	MR (***n*** = 28)	FFB (***n*** = 27)	***p***-value
**6 MWT, mean (sd)**	425 (44)	437 (80)	0.50
**Stair climb test, mean (sd)**	14.0 (3.6)	14.4 (4.1)	0.69
**Peak quadriceps force (N/BMI), mean (sd)**	1.38 (0.48)	1.67 (0.55)	**0.03**
**EQ-5D-5L utility, median (IQR)**	1.0 (0.8-1.0)	0.9 (0.8-1.0)	0.32
**EQ-5D-5L VAS, median (IQR)**	82.5 (80.0-95.0)	90.0 (75.0-95.0)	0.63
**Lysholm Total score grouped**			0.94
**Excellent**	10 (35.7)	9 (33.3)	
**Good**	4 (14.3)	5 (18.5)	
**Fair**	8 (28.6)	8 (29.6)	
**Poor**	6 (21.4)	5 (18.5)	

*Abbreviations*: *MR* measured resection, *FFB* Flexion First Balancer, *6MWT* 6-min walking test, *sd* standard deviation, *IQR* interquartile range, *N/BMI* newton / body mass index, *VAS* visual analogue scale, N.A.

For the total group (MR and FFB combined), no correlations were seen between the change in joint line height and the medial (r = 0.16, *p* = 0.24), lateral (r = − 0.08, *p* = 0.57) or total laxity (r = 0.17, *p* = 0.22). Therefore, subsequent correlation analyses were only performed between the change in joint line height (not for laxity) and the functional outcomes. A significant moderate negative correlation was found between joint line elevation and quadriceps peak force (r = − 0.29, *p* < 0.05). No significant correlations were found with the 6MWT (r = 0.23, *p* = 0.10), SCT (r = 0.05, *p* = 0.70), or Lysholm score (r = 0.09, *p* = 0.52).

## Discussion

The main finding of this study illustrated no difference in mid-flexion laxity between the new FFB technique and the MR technique in TKA. The previously established joint line difference between FFB and MR after surgery was reconfirmed but no correlation between joint line elevation and laxity was found. The reconstructed joint line resulted in a higher quadriceps peak force for patients in the FFB technique group. This was emphasized by the negative moderate correlation found between joint line elevation and quadriceps peak force. All other functional outcome measures were comparable between the two groups.

The mid-flexion laxity (30 degrees flexion) found in our study was comparable with previously reported coronal laxity after TKA in 0 to 30 degrees flexion [20, 21]. Although cadaver studies clearly demonstrated the effect of joint line height on mid-flexion laxity [3, 4], the difference in joint line change between the MR and FFB group in the current study did not result in a difference in mid-flexion laxity. A possible explanation for this discrepancy might be that the difference in joint line height between the two groups in the current study was too small. Cross and colleagues showed that a 2 mm joint line elevation resulted in minor mid-flexion laxity and 4 mm elevation in clear mid-flexion laxity [3]. The difference in joint line position between our two groups was only 3 mm. This hypothesis was recently confirmed in a study by Minoda and colleagues, who showed no difference in mid-flexion laxity after a 2 mm joint line elevation during TKA surgery [22]. A second explanation could be that the results found in cadaver studies are not viable for in vivo testing. Patients need to fully relax the examined leg during the varus and valgus testing. Possibly, in conscious patients a compensatory effect of muscle tension is present. This assumption is underlined by the finding that ligament laxity increases after general anesthesia [23]. Thus, the limited amount of joint line elevation and the active muscle tension might have counter-acted possible mid-flexion laxity.

With regards to the secondary outcomes, only a significant higher peak quadriceps force was found for the FFB group compared to the MR group. Since the study lacks pre-operative functional test results this could in theory be a confounding result. But since other functional test were comparable between the two matched groups, we expect comparable fitness levels between the two groups. The higher peak quadriceps force can be explained by the increased joint line and the compensatory reduced posterior condylar offset in the MR group. The posterior condylar offset acts as a lever arm for the quadriceps muscle and if this arm is reduced this negatively affects the quadriceps strength. The FFB technique restores the medial condylar offset and therefore does not affect the quadriceps strength. This was also supported by the finding of a significant negative correlation between joint line elevation and peak quadriceps force in the total cohort. The outcomes of the functional tests and questionnaires revealed no differences between the groups. Our findings for 6MWT, SCT and peak quadriceps force were comparable to the literature [24–26]. The hypothesis that a reconstructed joint line would result in less laxity, thus improving relevant clinical functional outcome was not proven. Moreover, no correlation was found between joint line elevation and 6MWT and SCT. A possible factor might be the previously stated minimal difference in joint line height between the two groups, which resulted in comparable laxity results. Since our sample size calculation was based on medial and lateral laxity as the primary outcome, group sizes might have been too small for the functional outcome tests to show any differences between the two groups. Nevertheless, the negative correlation between joint line elevation after TKA and postoperative quadriceps force seems clinically relevant and should lead to extra care taken on joint line reconstruction during TKA placement.

The FFB technique has been a regular part of our practice for several years. Multiple studies hinted to the possible benefits of this new technique. With the FFB technique it is safely possible to reconstruct the joint line however this does not result in better PROMs [7]. Hence the current study, that focused on coronal laxity and functional outcome. With exception for a higher peak quadriceps force in the FFB group no other benefits could be identified. It can therefore be concluded that the FFB technique is safe to use and is without negative side effects. However, demonstrating actual improvements in clinical outcome for patients after TKA is difficult. Apart from increased peak quadriceps force, due to a better reconstructed joint line, no other benefits for the patient could be identified. As with all ligament balancing systems, extreme deformities and insufficient collateral ligaments are a contraindication for this technique [27].

There are some limitations regarding this research. This cross-sectional study compared two techniques without blinding and randomization. And although the results in this study were prospectively acquired, the preoperative values were retrospectively collected. In 2015 we started using the FFB technique exclusively for TKA. Therefore, no direct comparison between the two groups has been possible. There is very little difference shown in follow-up between the two cohorts but short follow-up time (15 months for 1 patient) could be an limitation. However, since both cohorts had more than 12 months of follow-up an effect on the outcome measures is not expected as was concluded in a recently published systematic review by Ramkumar and colleagues [28]. Secondly, the power-calculation was performed for varus-valgus laxity, which might have resulted in underpowered results for the functional tests and questionnaires. But early studies concerning functional outcome with similar group sizes were however able to find significant differences between groups so it could be argued that group sizes were sufficient [26, 29]. Thirdly, due to the nature of this cross-sectional study preoperative quadriceps force is missing for patients which might be a confounder for the reported significant difference between the two groups. Finally, with regards to validity for stress radiographs, for anterior-posterior instability of the knee this is well documented however, for coronal laxity this information is still missing. Since these stress radiographs are widely used in preoperative as well as research settings, this should be addressed in future research.

## Conclusion

The FFB technique did not lead to less coronal laxity in the mid-flexion range compared to MR technique. Although peak quadriceps force was significantly higher for the FFB group no clinically relevant benefits could be identified for the patients with regards to functional outcome. Finally the negative correlation between joint line elevation after TKA and postoperative quadriceps force seems clinically relevant. The Flexion First gap balancing method might provide a more controlled joint line reconstruction in TKA although, small deviations in joint line seems to have no effect on functional outcome after TKA.

## Data Availability

The datasets used and/or analysed during the current study are available from the corresponding author on reasonable request.

## Abbreviations

6MWT: 6-min walking test
ASA: American Society of Anesthesiologists
BMI: Body mass index
FFB: Flexion First Balancer
MCL: Medial collateral ligament
MR: Measured resection
PROMs: Patient recorded outcome measures
SCT: Stair climbing test
TKA: Total knee arthroplasty
